# Synthesis, Characterization, and Antifungal Activity of Pyridine-Based Triple Quaternized Chitosan Derivatives

**DOI:** 10.3390/molecules23102604

**Published:** 2018-10-11

**Authors:** Lijie Wei, Yuan Chen, Wenqiang Tan, Qing Li, Guodong Gu, Fang Dong, Zhanyong Guo

**Affiliations:** 1 Key Laboratory of Coastal Biology and Bioresource Utilization, Yantai Institute of Coastal Zone Research, Chinese Academy of Sciences, Yantai 264003, China; ljwei@yic.ac.cn (L.W.); yuanchen@yic.ac.cn (Y.C.); wqtan@yic.ac.cn (W.T.); qli@yic.ac.cn (Q.L.); 2 University of Chinese Academy of Sciences, Beijing 100049, China; 3 Alliance Pharma, Inc., 17 Lee Boulevard, Malvern, PA 19355, USA; guguodong011@gmail.com

**Keywords:** chitosan, pyridine, quaternized, antifungal

## Abstract

In this study, a series of triple quaternized chitosan derivatives, including 6-*O*-[(2-hydroxy-3-trimethylammonium)propyl]-2-*N*-(1-pyridylmethyl-2-ylmethyl)-*N*,*N*-dimethyl chitosan chloride (**7**), 6-*O*-[(2-hydroxy-3-trimethylammonium)propyl]-2-*N*-(1-pyridylmethyl-3-yl- methyl)-*N*,*N*-dimethyl chitosan chloride (**8**), and 6-*O*-[(2-hydroxy-3-trimethylammonium)propyl]- 2-*N*-(1-pyridylmethyl-4-ylmethyl)-*N*,*N*-dimethyl chitosan chloride (**9**) were successfully designed and synthesized via reacting epoxypropyl trimethylammonium chloride with the *N*-pyridinium double quaternized chitosan derivatives. Detailed structural characterization was carried out using FT-IR and ^1^H-NMR spectroscopy, and elemental analysis. Besides, the activity of the triple quaternized chitosan derivatives against three common plant pathogenic fungi, *Watermelon fusarium*, *Fusarium oxysporum*, and *Phomopsis asparagi*, was investigated in vitro. The results indicated that the triple quaternized chitosan derivatives had enhanced antifungal activity when compared to double quaternized chitosan derivatives and chitosan, especially at 1.0 mg/mL, which confirmed the theory that the higher density of positive charge contributed to the antifungal activity. Moreover, **8** with an almost 99% inhibitory index showed the better antifungal activity against *Watermelon fusarium*. Moreover, the cytotoxicity of the products was also evaluated in vitro on 3T3-L1 cells and all the triple quaternized chitosan derivatives exhibited low cytotoxicity. These results suggested that triple quaternized chitosan derivatives may be used as good antifungal biomaterials.

## 1. Introduction

There are a large number of economic losses in agricultural production worldwide, especially in developing countries [1]. Plant pathogenic fungi have a negative effect on a substantial number of important fruits and vegetables, which limits the crop yield [2]. For example, Fusarium wilt, caused by *Fusarium oxysporum f*. sp. *Niveum*, is the most severe soilborne disease of watermelons worldwide, which is difficult to manage because the pathogen can survive for a long period of time in the soil as chlamydospores [3]. *Fusarium oxysporum* is one of the most damaging diseases affecting cucumber to limit production or decrease fruit quality in most areas of the world, which will cause the plant to develop necrotic lesions at the stem base, foliar wilting, yellowing of the lower leaves and eventually death [4,5]. *Phomopsis asparagi* can cause stem blight, fern defoliation, and subsequent yield loss, particularly in humid areas [6]. Chemical pesticides have been extensively used to control the growth of these fungi [7]. However, pesticide residues give rise to a potential risk to the environment and human health [8]. Therefore, the search for a safe, efficacious, environmentally friendly, and natural alternatives is necessary in plant protection.

Chitosan is one of the most abundant natural polysaccharides and its antifungal activity against various groups of pathogenic fungi has drawn a lot of attention [9]. As the only alkaline polysaccharide in nature, chitosan is mainly obtained via the deacetylation of chitin under alkaline conditions [10,11,12]. The main chain of chitosan is mainly composed of glucosamine and *N*-acetylglucosamine [13]. In addition, chitosan has some unique characteristics, such as non-toxicity, biodegradability, and biocompatibility, which facilitate its emerging applications in food science, agriculture, medicine, pharmaceuticals, and textiles [14,15,16,17,18]. However, its poor water solubility under neutral and alkaline condition limits the widespread application of chitosan [19]. Chemical modification is one of the most effective methods used to improve the water solubility and the bioactivity of chitosan due to the presence of primary amino groups and hydroxyl groups in chitosan [20,21]. Therefore, several chemically modified functional derivatives have been prepared via carboxylation, quaternization, phosphorylation, and sulfation to improve the water solubility and bioactivity of chitosan [22,23,24,25].

Quaternary ammonium salts with low toxicity have been widely used in bandages, the solutions used for rinsing open wounds, preoperative disinfectants, and paracellular transport of macromolecular drugs in medicine and pharmacy [26,27,28]. Various quaternized amines, including benzalkonium, pyridinium, and triethylammonium bromide, have very strong bioactivities including antibacterial, antifungal, and antiprotozoal activity [21,29]. Based on the superposition principle, quaternary ammonium salts have been introduced into various polymers to improve their bioactivity. For example, quaternized chitosan derivatives have better antifungal activity than *N*-substituted and Schiff base chitosan derivatives, and it has been proposed that the antifungal activity is affected by the positive charge of the quaternized chitosan [21].

In this paper, a series of triple quaternized chitosan derivatives were successfully synthesized and their antifungal activity was investigated in vitro. The preparation of the double quaternized derivatives used in this paper has already been previously published, but without reporting their antifungal activities [30]. Besides, it has been reported that chitosan nanoparticles fabricated from the fatty acid and epoxy propyl trimethyl ammonium chloride (GTMAC) are effective in a liver-target deliver system of insulin [31]. GTMAC, as an important cationic etherifying agent, has been introduced at the C-6-OH of chitosan in this study. The chemical structures of the derivatives were characterized using FT-IR and ^1^H-NMR spectroscopy, and elemental analysis. Furthermore, the activities of the chitosan derivatives against *Watermelon fusarium*, *Fusarium oxysporum*, and *Phomopsis asparagi* were investigated in this study, respectively.

## 2. Results

### 2.1. Structure of the Chitosan Derivative

**Figure 1:**
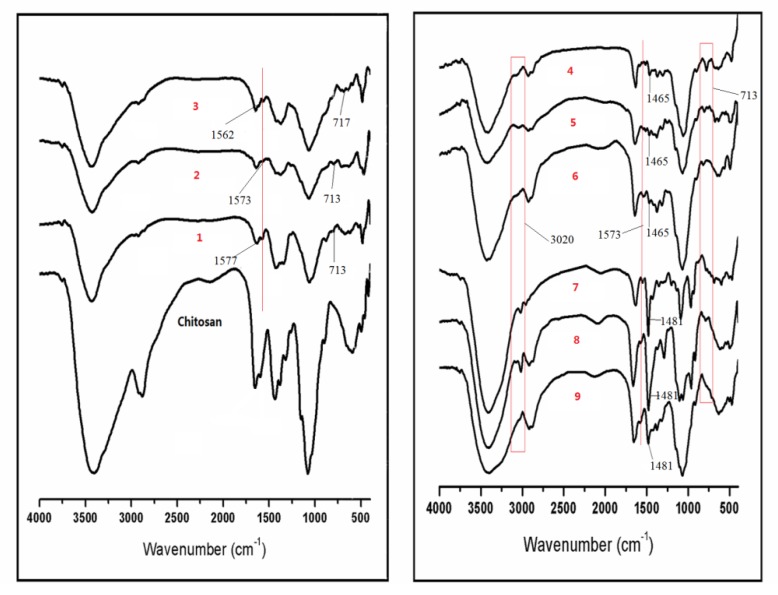
FT-IR spectra of chitosan and chitosan derivatives.

The structures of the quaternized chitosan derivatives were determined using FT-IR and ^1^H-NMR spectroscopy, and elemental analysis. The FT-IR spectra of chitosan and quaternized chitosan derivatives are shown in Figure 1. The spectrum of chitosan shows that the saccharide mainly contains the following characteristic bands: ν (O-H) or ν (N-H) 3405 cm^−1^, ν (C-H) 2881 cm^−1^, ν (amide I band) 1654 cm^−1^, ν (-NH2) 1596 cm^−1^, δ (C-H) 1434 and 1384 cm^−1^, ν (amide III band) 1319 cm^−1^, δ (O-H) 1261 cm^−1^, ν (C-O) 1076 cm^−1^, and 898 cm^−1^ which indicates the β glycosidic bond. In the *N*-methylpyridine chitosan derivatives, the new peaks at 1577 cm^−1^, 1573 cm^−1^ and 1562 cm^−1^ were assigned to the pyridine rings in **1**, **2** and **3**, respectively. Moreover, **1**, **2** and **3** all had a new peak at about 713 or 717 cm^−1^ corresponding to the pyridine ring with a different substitution position. After being quaternized, except for the absorption peaks of the pyridine ring at 3020, 1573 and 713 cm^−1^, there was a new peak at about 1465 or 1469 cm^−1^, which was assigned to the absorption of N-CH_3_ in **4**, **5** and **6**. When GTMAC was introduced onto the polysaccharide ring, a new strong peak at 1481 cm^−1^ due to the absorption of the trimethyl group in **7**, **8** and **9** was observed. The above results demonstrated that the triple quaternized chitosan derivatives were obtained.

**Figure 2:**
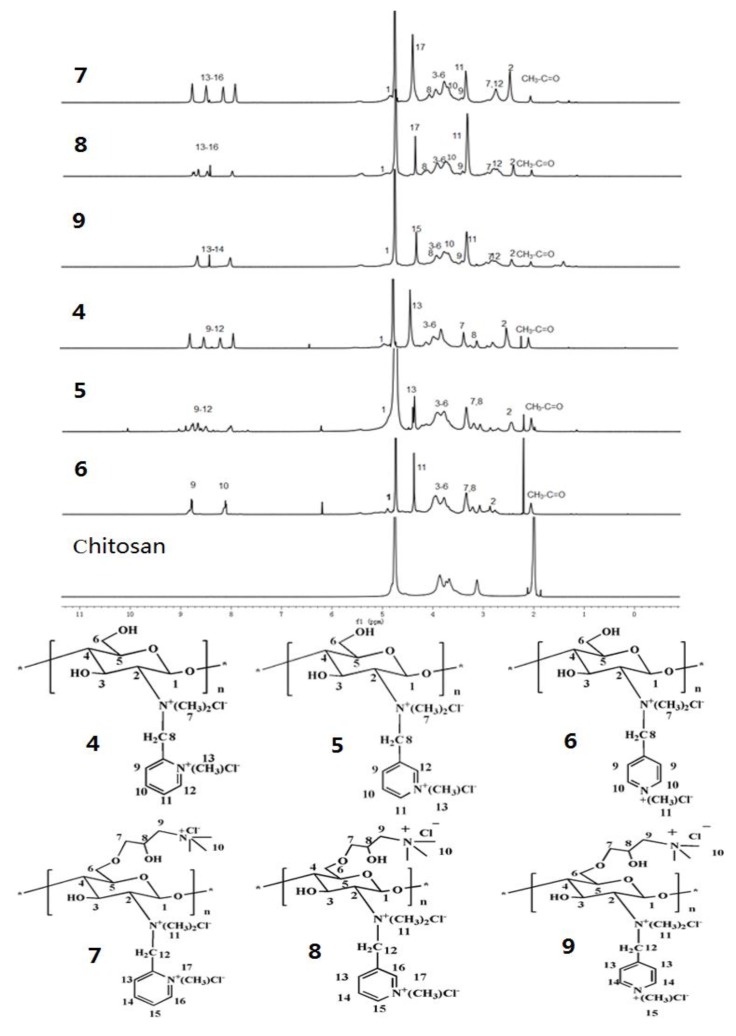
^1^H-NMR spectra of chitosan, **4**, **5**, **6**, **7**, **8** and **9**.

Figure 2 shows the ^1^H-NMR spectra recorded for chitosan and all the quaternized chitosan derivatives. Each group of absorption peaks were marked in Figure 2. The absorption peaks of protons in the glucose skeleton of chitosan were observed at δ = 5.1–3.8 ppm. In **4**, **5** and **6**, the characteristic resonance of N-CH_3_ at C7 was observed at δ = 3.3 ppm. Besides, the peaks at δ = 4.4, 4.3 and 4.3 ppm corresponded to the methyl protons grafted to nitrogen on the pyridine ring, respectively. Meantime, the peaks at δ = 8.0–9.3 ppm corresponded to the pyridine ring protons. In addition to the above characteristic absorption peaks, there was a new peak δ = 3.7 ppm, which was assigned to the trimethyl group in the 2-hydroxy-3-(trimethylammonium)propyl moiety in **7**, **8** and **9**. The ^1^H-NMR spectra confirmed that the triple quaternized chitosan derivatives were successfully synthesized.

### 2.2. Antifungal Activity

Chitosan has poor water solubility, but all the quaternized chitosan derivatives showed good solubility in neutral water because the quaternized amine salts acted as hydrophilic moieties to significantly improve the solubility of chitosan [32]. We used water-soluble low molecular chitosan in the antifungal activity study. Herein, we investigated the antifungal activity of chitosan, the intermediate double quaternized chitosan derivatives, and triple quaternized chitosan derivatives against three common plant-threatening fungi, *Watermelon fusarium*, *Fusarium oxysporum*, and *Phomopsis asparagi*, in vitro by measuring the growth rate of mycelium. Carbendazim was used as the positive control in this study. All the samples were prepared at 0.1–1.0 mg/mL concentration at room temperature. Their antifungal indices and rules were discussed as follows.

**Figure 3:**
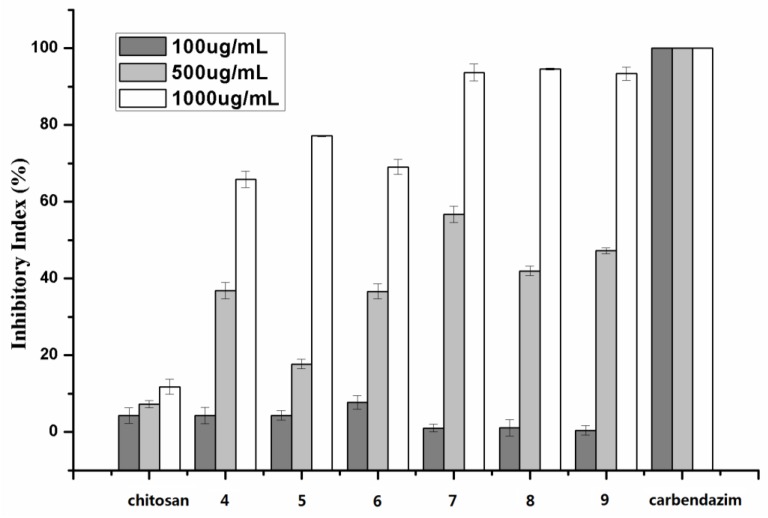
The antifungal activity of chitosan and chitosan derivatives against *Fusarium oxysporum.*

The antifungal activity of chitosan and all the quaternized chitosan derivatives against *Fusarium oxysporum* was investigated in vitro. As shown in Figure 3, chitosan and the quaternized chitosan derivatives all exhibited antifungal activity against *Fusarium oxysporum*. Moreover, the inhibitory indices of all the samples were enhanced upon increasing the concentration. The inhibitory indices of chitosan, **4**, **5**, **6**, **7**, **8** and **9** were 11.8%, 65.8%, 77.1%, 69.1%, 93.7%, 94.5% and 93.3% at 1.0 mg/mL, respectively. 

**Figure 4:**
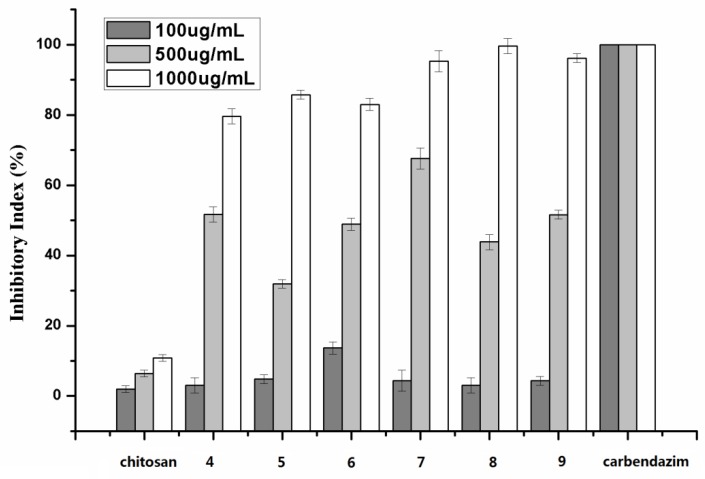
The antifungal activity of chitosan and chitosan derivatives against *Watermelon fusarium*.

All the quaternary ammonium salts exhibited enhanced antifungal activity when compared with chitosan. Besides, the triple quaternized chitosan derivatives (**7**, **8** and **9**) showed higher inhibitory indices than the double quaternized chitosan derivatives (**4**, **5** and **6**). On the one hand, the higher density of the positive charge will contribute to the antifungal activity [33]. On the other hand, the introduced aliphatic alcohol in **7**, **8** and **9** may be another reason for the enhanced antifungal activity because it has been reported that the antifungal activity of chitosan derivatives can be improved using a long length aliphatic alcohol substituent [19]. Moreover, the antifungal activity of the compound with the nitrogen atom in the 3 position of the pyridine ring was better than that observed for the compound with the nitrogen atom in the 2/4 position. The delocalization of the positive charge on the *N*-pyridinium group at the 2 and 4 position was more significant than that at the 3 position [30]. Figure 4 shows the inhibitory indices of chitosan, **4**, **5**, **6**, **7**, **8** and **9** against *Watermelon fusarium*. The results were similar to the antifungal activity observed against *Fusarium oxysporum*. First, all the samples showed antifungal activity against *Watermelon fusarium*. Secondly, the inhibitory indices of chitosan and the chitosan derivatives increased upon increasing the concentration. In addition, the inhibitory indices of chitosan, **4**, **5**, **6**, **7**, **8** and **9** were 10.8%, 80.0%, 85.8%, 83.0%, 96.3%, 99.6% and 96.2% at 1.0 mg/mL, respectively. It was apparent that the triple quaternized chitosan derivatives, including **7**, **8** and **9**, with inhibitory indices >95.0% had enhanced antifungal properties when compared to the double quaternized chitosan derivatives. The observation could be attributed to the higher positive charge density and the aliphatic alcohol group introduced into the chitosan molecule in triple quaternized chitosan derivatives. It is worth noting that 9 with an inhibitory index of 99% almost completely inhibited the growth of the fungi studied.

**Figure 5:**
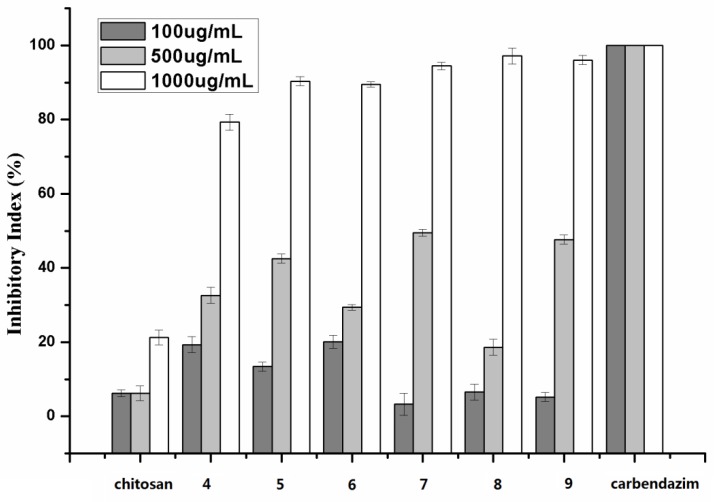
The antifungal activity of chitosan and chitosan derivatives against *Phomopsis asparagi*.

As shown in Figure 5, chitosan and the quaternized chitosan derivatives exhibited antifungal activity against *Phomopsis asparagi* at all the tested concentrations. Similar to the antifungal activity against *Fusarium oxysporum*, and *Watermelon fusarium*, the inhibitory indices increased upon increasing the concentration. Compounds **4**, **5**, **6**, **7**, **8** and **9** with inhibitory indices 79.3%, 90.3%, 89.5%, 94.5%, 97.2% and 96.1% at 1.0 mg/mL respectively exhibit much stronger antifungal activity than chitosan (inhibitory index = 21.0% at 1.0 mg/mL). Besides, the antifungal activity of the samples decreased in the order of **5** > **6** > **4**; **8** > **9** > **7**, especially at 1.0 mg/mL. The different positions of the nitrogen atom on the pyridine ring impact the antifungal activity.

Based on the results mentioned above, the antifungal activity of the triple quaternized chitosan derivatives was much stronger than that of chitosan and the double quaternized chitosan derivatives, especially at 1.0 mg/mL. This was in accordance with the theory that the higher density of positive charge will contribute to the antifungal activity. On the one hand, the positive charge will interact with anionic components, such as glucan, mannan, proteins, and lipids, to form an impervious layer around the cell, which will prevent the nutrient exchange in the cells [17]. On the other hand, this interaction can damage the cell wall and cause the leakage of the cell constituents [17]. In addition, the long length aliphatic alcohol introduced in the triple quaternized chitosan derivatives can contribute to the antifungal activity [19]. Moreover, the positive charged location on the pyridine ring of the quaternized chitosan derivatives were also factors that affected the antifungal activity of the samples. Test results showed that compounds with 3 position of the nitrogen atom on the pyridine ring had stronger antifungal activity than those with 2/4 position.

### 2.3. Cytotoxicity Analysis

**Figure 6:**
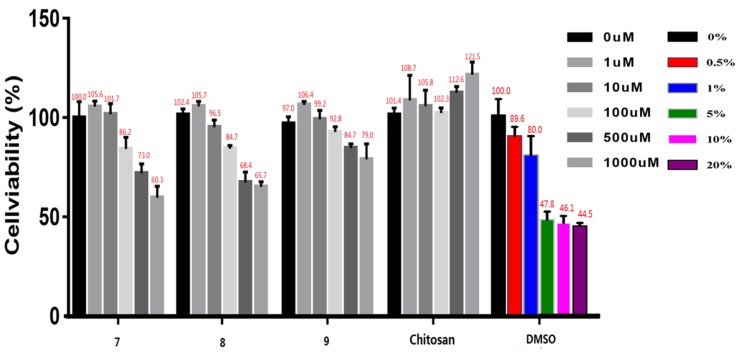
The cytotoxicity of chitosan and chitosan derivatives on 3T3-L1 cells.

The cytotoxicity of chitosan and quaternized chitosan derivatives on 3T3-L1 cells at different concentrations was evaluated using an MTT assay [34]. Figure 6 shows that the growth of the 3T3-L1 cells was subjected to different degrees of inhibition after 24 h of treatment using chitosan and the triple quaternized chitosan derivatives at different concentrations. As shown in Figure 6, DMSO was used as the positive control at the various volume fraction. The cell viability value was only 47.8% at 5% of the volume fraction. Besides, the cell viability values of the cells treated with **7**, **8** and **9** decreased upon increasing the concentration, which demonstrated that the cytotoxicity of the triple quaternized chitosan derivatives was dose-dependent. Besides, no cytotoxicity was observed for the 3T3-L1 cells incubated with pristine chitosan at all the testing concentrations studied. Compound **9** with a cell viability index of >82.0% at 1000 μM indicated good biocompatibility. Upon decreasing the concentration, the cell viability increased gradually. Compounds **7** and **8** had a cell viability of >88.2% at 100 μM. In consideration of the excellent antifungal activity and low cytotoxicity of the triple quaternized chitosan derivatives, the products may be considered to have good biocompatibility.

## 3. Materials and Methods

### 3.1. Materials

Chitosan (MW 2.00 × 10^5^, the degree of deacetylation 82.6%) was purchased from Qingdao Baicheng Biochemical Corp. (Qingdao, China). 2-Pyridinecarboxaldehyde (product code P105912), 3-pyridinecarboxaldehyde (product code P106877), 4-pyridinecarboxaldehyde (product code P105913), sodium borohydride (NaBH_4_) (product code S108355), *N*-methyl-2-pyrrolidone (NMP) (product code M103247), sodium iodide (NaI) (product code S105954), and sodium hydroxide (NaOH) (product code S111501) were purchased from Aladdin Industrial Corp. (Shanghai, China). The other reagents were purchased from Sinopharm Chemical Reagent Co., Ltd. (Shanghai, China).

### 3.2. Analytical Methods

FT-IR spectra were measured from 4000 to 400 cm^−1^ on a Jasco-4100 spectrophotometer (JASCO Co., Ltd., Shanghai, China) at 25 °C with KBr disks. ^1^H-NMR spectra were recorded on a Bruker AVIII 500 spectroscopy (Bruker Tech. and Serv. Co., Ltd., Beijing, China), using D_2_O as solvent with tetramethysilane (TMS) as an internal standard. Chemical shift values were given in δ (ppm). The elemental analyses (C, H, and N) were performed on a Vario EL III (Elementar, Langenselbold, Germany). The Degree of Substitution (DS) of chitosan derivatives was calculated based on the percentages of carbon and nitrogen. The results were processed and reported as mean ± SD by the computer programs Excel (Microsoft, California, WA, USA), OriginPro 8 (OriginLab, Massachusetts, MA, USA), and MestReNova (Mestrelab Research S.L., Asturias, Spain).

### 3.3. Synthesis of Pyridine-Based Double Quaternized Chitosan Derivatives ***4***, ***5*** and ***6***

**Scheme 1:**
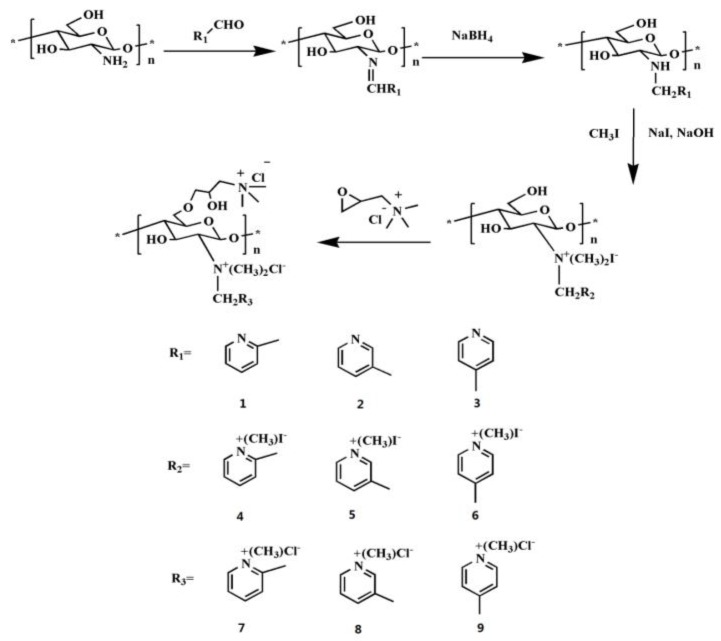
The synthesis of triple quaternized chitosan derivatives.

The preparation of triple quaternized chitosan derivatives is shown in Scheme 1. The double quaternary ammonium salts chitosan derivatives were prepared according the previous work [30]. In brief, 1.6 g of chitosan were dispersed into 20.0 mL of ethanol and 50.0 mL of 1.0% acetic acid solution in flask at 25 °C. Then 30.0 mmol of various pyridylaldehydes (2-pyridinecarboxaldehyde 2.9 mL; 3-pyridinecarboxaldehyde 2.8 mL; 4-pyridinecarboxaldehyde 2.9 mL) were added dropwise with stirring at 25 °C respectively. Then another 20.0 mL of ethanol were added to disperse the solution. After 4 h, 1.8 g of NaBH_4_ were added and the system was continuously reacted for another 4 h. Then the solution was precipitated into excess acetone. The precipitate was filtrated and washed with acetone. The *N*-methylpyridine chitosan derivatives (**1**, **2** and **3**) were obtained after drying at 60 °C for 24 h respectively. Then 0.5 g of the above synthesized *N*-methylpyridine chitosan were dispersed into 30.0 mL of NMP for 12 h at 25 °C respectively, followed by 0.2 mL of NaOH solution (1 M), 1.5 g of NaI, and 4.0 mL of CH_3_I were added respectively, and the reaction was carried out at 60 °C for 4 h with reflux. Then the solution was precipitated by excess acetone and the precipitate was filtered and washed with acetone. The double quaternized chitosan derivatives including **4**, **5** and **6** were obtained by drying at 60 °C for 24 h respectively. **4**: yield: 86.3%; DS: 50.4%; **5**: yield: 84.6%; DS: 53.2%; **6**: yield: 86.2%; DS: 49.0% (Table 1).

### 3.4. Synthesis of Triple Quaternized Chitosan Derivatives ***7***, ***8*** and ***9***

To a round bottom flask equipped with a cooling system, 0.2 g of the above double quaternized chitosan derivative were added into 30.0 mL of isopropanol, respectively. Then 0.5 g of NaOH solution (40% wt) were added into the system and the reaction was stirred at 25 °C for 1 h. Then 1.8 g of GTMAC were dropped, and the mixture was heated at 70 °C for 6 h. The product was poured into extra ethanol, and the precipitate was filtered and washed with ethanol. The unreactive reagents were extracted in a Soxhlet apparatus with a mixture of ethanol and acetone for 48 h. Then triple quaternized chitosan derivatives (**7**, **8** and **9**) were obtained by freeze-drying overnight in vacuum respectively. **7**: yield: 78.3%; DS: 90.3%; **8**: yield: 80.6%; DS: 92.6%; **9**: yield: 76.2%; DS: 91.4% (Table 1).

### 3.5. Antifungal Assay

The antifungal assay was evaluated against *Watermelon fusarium*, *Fusarium oxysporum*, and *Phomopsis asparagi* in vitro by measuring the growth rate of mycelium according to Guo’s method [29]. Briefly, the compounds (chitosan and all quaternized chitosan derivatives) were dissolved in the distilled water at the concentration of 5.0 mg/mL at room temperature. Then, the test sample solution was added to the sterilized potato dextrose agar (PDA) medium to get a final concentration of 0.1, 0.5, and 1.0 mg/mL respectively; then the mixture was poured into the sterilized Petri dishes (9.0 cm). Same volume of distilled water substituting samples was poured into a control plate. Finally, the fungi mycelia disk with a diameter of 5.0 mm was placed in the center of the PDA Petri dishes and incubated at 27 °C for 2–3 days. When the diameter of the fungi mycelium reached the edges of the control plate (without the sample), the inhibitory index was calculated as follows: Inhibitory index (%) = (1 − Da/Db) × 100, where Da is the diameter of the growth zone in the test plates, and Db is the diameter of the growth zone in the control plate. The experiment was performed in three replicates. And the data were averaged and expressed as means ± SD (*n* = 3). Scheffe’s multiple range test was used to evaluate the inhibitory indices difference in antifungal tests. The level of *p* < 0.05 was regarded as statistically significant.

### 3.6. Cytotoxicity Assay

The cytotoxicity of chitosan and all the quaternized chitosan derivatives was investigated based on an in vitro MTT assay [34]. Briefly, 3T3-L1 cells were seeded on 96-well flat-bottom culture plates at a density of 1.0 × 10^5^ cells per well and incubated at 37 °C in a humidified 5% CO_2_ balanced-air incubator for 24 h. Then, the cells were treated with the compounds at different concentrations (1.0, 10.0, 100.0, 500.0, and 1000.0 μM, respectively) and the untreated cells were used as the control. After incubation at 37 °C for 24 h, the supernatants were carefully removed, then 10.0 μL of a 5.0 mg/mL of MTT stock solution and 90.0 μL of FBS-free medium were added into each well. After incubation for 4 h, the formazan crystals dissolved by MTT stopping buffer (10% sodium dodecyl sulfate and 0.01 M hydrochloric acid) were added into each well. The optical density at 550 nm was detected by an Infinite M200 Pro spectrophotometer (Tecan, Männedorf, Switzerland). The cell viability was recorded using the following formula: Cell viability (%) = *A_test_*/*A_control_* × 100, Where *A_test_* and *A_control_* are the absorbance values of the test and the control well, respectively.

## 4. Conclusions

In this paper, a series of triple quaternized chitosan derivatives were successfully designed, synthesized, and characterized. The antifungal activity against three kinds of phytopathogens, *Watermelon fusarium*, *Fusarium oxysporum*, and *Phomopsis* *asparagi*, were estimated using in vitro hyphal measurements. All the triple quaternized chitosan derivatives exhibited enhanced antifungal activity when compared with chitosan and the double quaternized chitosan derivatives. The higher density of positive charge and the long length aliphatic alcohol contributed to the antifungal activity. In addition, all the triple quaternized chitosan derivatives exhibited good biocompatibility as evaluated by an MTT assay against 3T3-L1 cells at 1–1000 μM, which could be used as good antifungal biomaterials. The relationship between the structure and the activity needs further study in the future.

## Figures and Tables

**Table 1:** The elemental analyses, yields, and the degrees of substitution of chitosan derivatives, and the degree of deacetylation of chitosan.

Compounds	Yields (%)	Elemental Analyses (%)			Degrees of Substitution (%)	Deacetylation (%)
		C	N	C/N		
Chitosan		43.42	7.98	5.44		82.6
**4**	86.3	34.17	5.60	6.10	50.4	
**5**	84.6	30.83	5.03	6.13	53.2	
**6**	86.2	33.01	5.42	6.09	49.0	
**7**	78.3	34.78	5.89	5.91	90.3	
**8**	80.6	39.22	6.61	5.93	92.6	
**9**	76.2	40.63	6.87	5.91	91.4	
